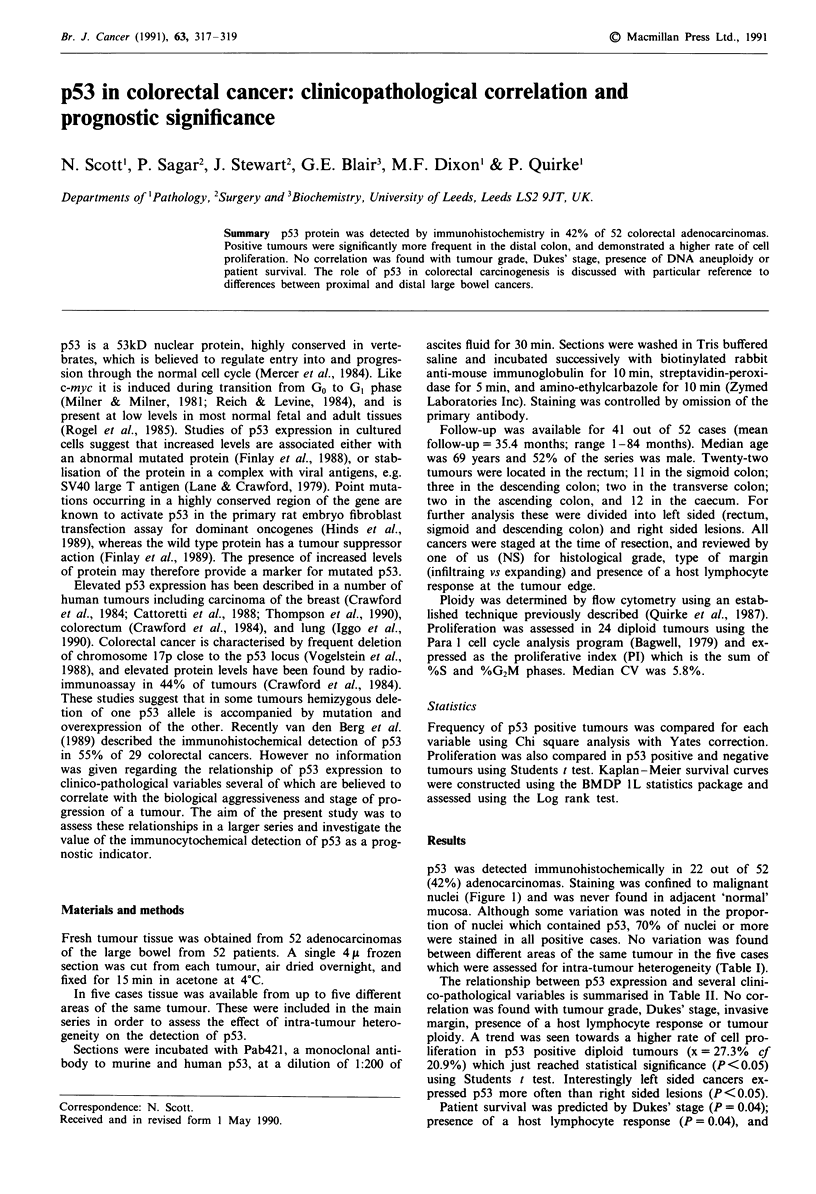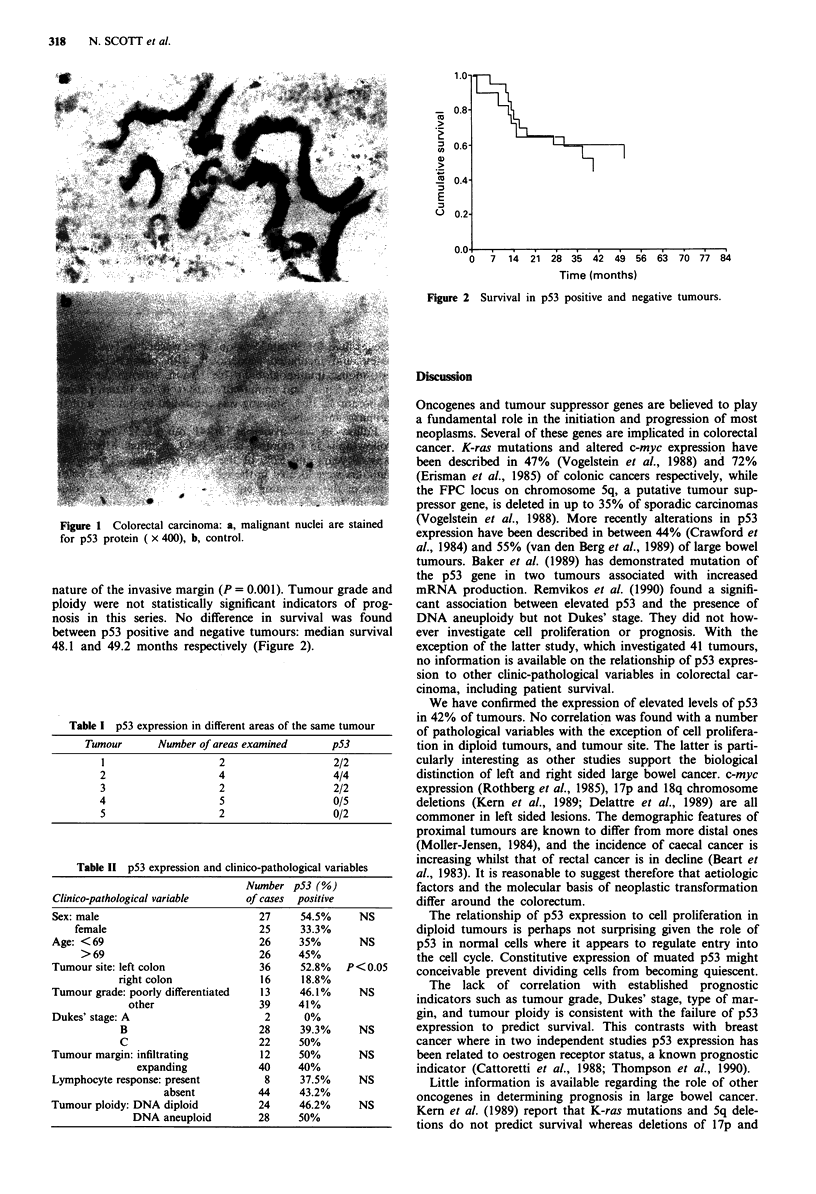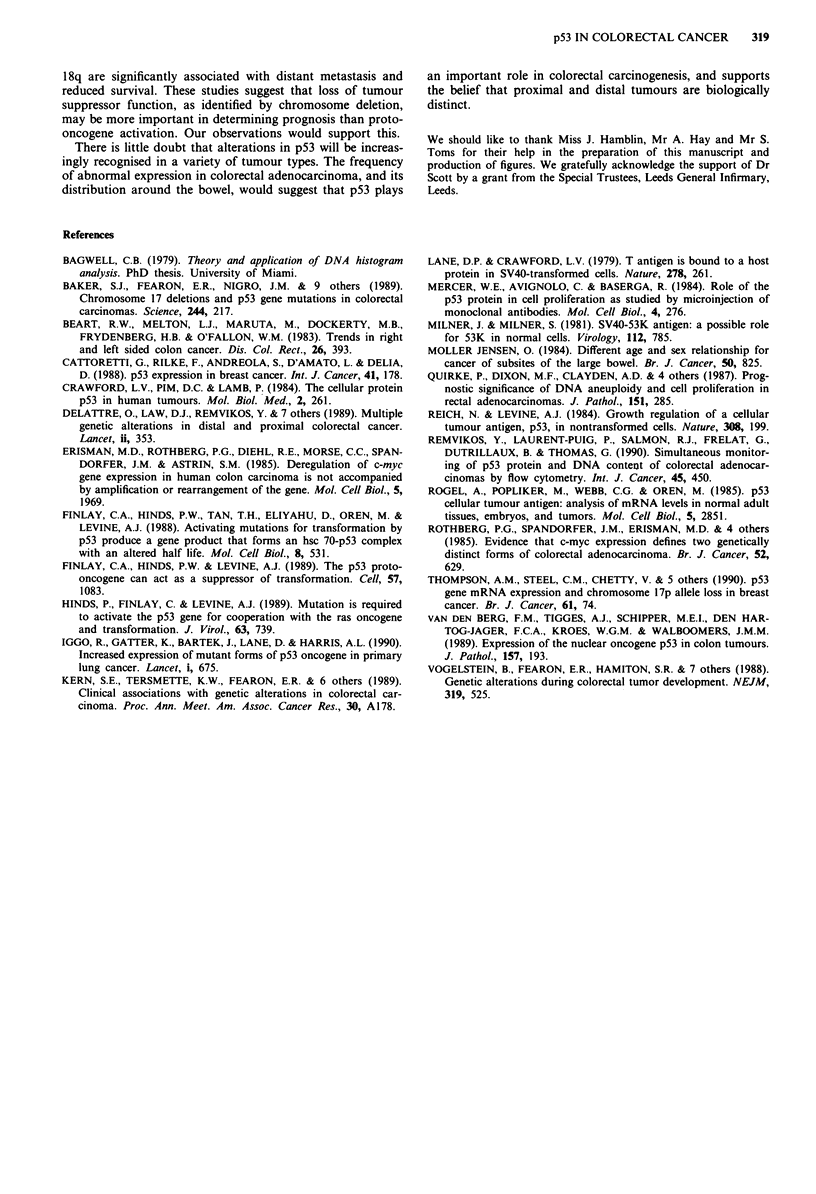# p53 in colorectal cancer: clinicopathological correlation and prognostic significance.

**DOI:** 10.1038/bjc.1991.74

**Published:** 1991-02

**Authors:** N. Scott, P. Sagar, J. Stewart, G. E. Blair, M. F. Dixon, P. Quirke

**Affiliations:** Department of Pathology, University of Leeds, UK.

## Abstract

**Images:**


Br. J. Cancer (1991), 63, 317–319 © Macmillan Press Ltd., 1991

### p53 in colorectal cancer: clinicopathological correlation and prognostic significance

N. Scott[1], P. Sagar[2], J. Stewart[2], G.E. Blair[3], M.F. Dixon[1] & P. Quirke[1]

*Departments of [1]Pathology, [2]Surgery and [3]Biochemistry, University of Leeds, Leeds LS2 9JT, UK.*

**Summary** p53 protein was detected by immunohistochemistry in 42% of 52 colorectal adenocarcinomas. Positive tumours were significantly more frequent in the distal colon, and demonstrated a higher rate of cell proliferation. No correlation was found with tumour grade, Dukes' stage, presence of DNA aneuploidy or patient survival. The role of p53 in colorectal carcinogenesis is discussed with particular reference to differences between proximal and distal large bowel cancers.

p53 is a 53kD nuclear protein, highly conserved in vertebrates, which is believed to regulate entry into and progression through the normal cell cycle (Mercer *et al.*, 1984). Like c-*myc* it is induced during transition from $G_0$ to $G_1$ phase (Milner & Milner, 1981; Reich & Levine, 1984), and is present at low levels in most normal fetal and adult tissues (Rogel *et al.*, 1985). Studies of p53 expression in cultured cells suggest that increased levels are associated either with an abnormal mutated protein (Finlay *et al.*, 1988), or stablisation of the protein in a complex with viral antigens, e.g. SV40 large T antigen (Lane & Crawford, 1979). Point mutations occurring in a highly conserved region of the gene are known to activate p53 in the primary rat embryo fibroblast transfection assay for dominant oncogenes (Hinds *et al.*, 1989), whereas the wild type protein has a tumour suppressor action (Finlay *et al.*, 1989). The presence of increased levels of protein may therefore provide a marker for mutated p53.

Elevated p53 expression has been described in a number of human tumours including carcinoma of the breast (Crawford *et al.*, 1984; Cattoretti *et al.*, 1988; Thompson *et al.*, 1990), colorectum (Crawford *et al.*, 1984), and lung (Iggo *et al.*, 1990). Colorectal cancer is characterised by frequent deletion of chromosome 17p close to the p53 locus (Vogelstein *et al.*, 1988), and elevated protein levels have been found by radioimmunoassay in 44% of tumours (Crawford *et al.*, 1984). These studies suggest that in some tumours hemizygous deletion of one p53 allele is accompanied by mutation and overexpression of the other. Recently van den Berg *et al.* (1989) described the immunohistochemical detection of p53 in 55% of 29 colorectal cancers. However no information was given regarding the relationship of p53 expression to clinico-pathological variables several of which are believed to correlate with the biological aggressiveness and stage of progression of a tumour. The aim of the present study was to assess these relationships in a larger series and investigate the value of the immunocytochemical detection of p53 as a prognostic indicator.

### Materials and methods

Fresh tumour tissue was obtained from 52 adenocarcinomas of the large bowel from 52 patients. A single 4 μ frozen section was cut from each tumour, air dried overnight, and fixed for 15 min in acetone at 4°C.

In five cases tissue was available from up to five different areas of the same tumour. These were included in the main series in order to assess the effect of intra-tumour heterogeneity on the detection of p53.

Sections were incubated with Pab421, a monoclonal antibody to murine and human p53, at a dilution of 1:200 of ascites fluid for 30 min. Sections were washed in Tris buffered saline and incubated successively with biotinylated rabbit anti-mouse immunoglobulin for 10 min, streptavidin-peroxidase for 5 min, and amino-ethylcarbazole for 10 min (Zymed Laboratories Inc). Staining was controlled by omission of the primary antibody.

Follow-up was available for 41 out of 52 cases (mean follow-up = 35.4 months; range 1–84 months). Median age was 69 years and 52% of the series was male. Twenty-two tumours were located in the rectum; 11 in the sigmoid colon; three in the descending colon; two in the transverse colon; two in the ascending colon, and 12 in the caecum. For further analysis these were divided into left sided (rectum, sigmoid and descending colon) and right sided lesions. All cancers were staged at the time of resection, and reviewed by one of us (NS) for histological grade, type of margin (infiltraing *vs* expanding) and presence of a host lymphocyte response at the tumour edge.

Ploidy was determined by flow cytometry using an established technique previously described (Quirke *et al.*, 1987). Proliferation was assessed in 24 diploid tumours using the Para 1 cell cycle analysis program (Bagwell, 1979) and expressed as the proliferative index (PI) which is the sum of %S and $\%G_2M$ phases. Median CV was 5.8%.

### *Statistics*

Frequency of p53 positive tumours was compared for each variable using Chi square analysis with Yates correction. Proliferation was also compared in p53 positive and negative tumours using Students *t* test. Kaplan–Meier survival curves were constructed using the BMDP 1L statistics package and assessed using the Log rank test.

### Results p53 was detected immunohistochemically in 22 out of 52 (42%) adenocarcinomas. Staining was confined to malignant nuclei (Figure 1) and was never found in adjacent 'normal' mucosa. Although some variation was noted in the proportion of nuclei which contained p53, 70% of nuclei or more were stained in all positive cases. No variation was found between different areas of the same tumour in the five cases which were assessed for intra-tumour heterogeneity (Table I).

The relationship between p53 expression and several clinico-pathological variables is summarised in Table II. No correlation was found with tumour grade, Dukes' stage, invasive margin, presence of a host lymphocyte response or tumour ploidy. A trend was seen towards a higher rate of cell proliferation in p53 positive diploid tumours (x = 27.3% *cf* 20.9%) which just reached statistical significance ($P < 0.05$) using Students *t* test. Interestingly left sided cancers expressed p53 more often than right sided lesions ($P < 0.05$).

Patient survival was predicted by Dukes' stage ($P = 0.04$); presence of a host lymphocyte response ($P = 0.04$), and

Correspondence: N. Scott.
Received and in revised form 1 May 1990.

Figure 1 Colorectal carcinoma: a, malignant nuclei are stained for p53 protein ( × 400), b, control.

nature of the invasive margin ($P = 0.001$). Tumour grade and ploidy were not statistically significant indicators of prognosis in this series. No difference in survival was found between p53 positive and negative tumours: median survival 48.1 and 49.2 months respectively (Figure 2).

Table I p53 expression in different areas of the same tumour

| *Tumour* | *Number of areas examined* | *p53* |
|---|---|---|
| 1 | 2 | 2/2 |
| 2 | 4 | 4/4 |
| 3 | 2 | 2/2 |
| 4 | 5 | 0/5 |
| 5 | 2 | 0/2 |

Table II p53 expression and clinico-pathological variables

| *Clinico-pathological variable* | *Number of cases* | *p53 (%) positive* | |
|---|---|---|---|
| Sex: male | 27 | 54.5% | NS |
| female | 25 | 33.3% | |
| Age: <69 | 26 | 35% | NS |
| >69 | 26 | 45% | |
| Tumour site: left colon | 36 | 52.8% | $P<0.05$ |
| right colon | 16 | 18.8% | |
| Tumour grade: poorly differentiated | 13 | 46.1% | NS |
| other | 39 | 41% | |
| Dukes' stage: A | 2 | 0% | |
| B | 28 | 39.3% | NS |
| C | 22 | 50% | |
| Tumour margin: infiltrating | 12 | 50% | NS |
| expanding | 40 | 40% | |
| Lymphocyte response: present | 8 | 37.5% | NS |
| absent | 44 | 43.2% | |
| Tumour ploidy: DNA diploid | 24 | 46.2% | NS |
| DNA aneuploid | 28 | 50% | |

Figure 2 Survival in p53 positive and negative tumours.

### Discussion

Oncogenes and tumour suppressor genes are believed to play a fundamental role in the initiation and progression of most neoplasms. Several of these genes are implicated in colorectal cancer. *K-ras* mutations and altered c-*myc* expression have been described in 47% (Vogelstein *et al.*, 1988) and 72% (Erisman *et al.*, 1985) of colonic cancers respectively, while the FPC locus on chromosome 5q, a putative tumour suppressor gene, is deleted in up to 35% of sporadic carcinomas (Vogelstein *et al.*, 1988). More recently alterations in p53 expression have been described in between 44% (Crawford *et al.*, 1984) and 55% (van den Berg *et al.*, 1989) of large bowel tumours. Baker *et al.* (1989) has demonstrated mutation of the p53 gene in two tumours associated with increased mRNA production. Remvikos *et al.* (1990) found a significant association between elevated p53 and the presence of DNA aneuploidy but not Dukes' stage. They did not however investigate cell proliferation or prognosis. With the exception of the latter study, which investigated 41 tumours, no information is available on the relationship of p53 expression to other clinic-pathological variables in colorectal carcinoma, including patient survival.

We have confirmed the expression of elevated levels of p53 in 42% of tumours. No correlation was found with a number of pathological variables with the exception of cell proliferation in diploid tumours, and tumour site. The latter is particularly interesting as other studies support the biological distinction of left and right sided large bowel cancer. c-*myc* expression (Rothberg *et al.*, 1985), 17p and 18q chromosome deletions (Kern *et al.*, 1989; Delattre *et al.*, 1989) are all commoner in left sided lesions. The demographic features of proximal tumours are known to differ from more distal ones (Moller-Jensen, 1984), and the incidence of caecal cancer is increasing whilst that of rectal cancer is in decline (Beart *et al.*, 1983). It is reasonable to suggest therefore that aetiologic factors and the molecular basis of neoplastic transformation differ around the colorectum.

The relationship of p53 expression to cell proliferation in diploid tumours is perhaps not surprising given the role of p53 in normal cells where it appears to regulate entry into the cell cycle. Constitutive expression of muated p53 might conceivable prevent dividing cells from becoming quiescent.

The lack of correlation with established prognostic indicators such as tumour grade, Dukes' stage, type of margin, and tumour ploidy is consistent with the failure of p53 expression to predict survival. This contrasts with breast cancer where in two independent studies p53 expression has been related to oestrogen receptor status, a known prognostic indicator (Cattoretti *et al.*, 1988; Thompson *et al.*, 1990).

Little information is available regarding the role of other oncogenes in determining prognosis in large bowel cancer. Kern *et al.* (1989) report that K-*ras* mutations and 5q deletions do not predict survival whereas deletions of 17p and

18q are significantly associated with distant metastasis and reduced survival. These studies suggest that loss of tumour suppressor function, as identified by chromosome deletion, may be more important in determining prognosis than proto-oncogene activation. Our observations would support this.

There is little doubt that alterations in p53 will be increasingly recognised in a variety of tumour types. The frequency of abnormal expression in colorectal adenocarcinoma, and its distribution around the bowel, would suggest that p53 plays an important role in colorectal carcinogenesis, and supports the belief that proximal and distal tumours are biologically distinct.

We should like to thank Miss J. Hamblin, Mr A. Hay and Mr S. Toms for their help in the preparation of this manuscript and production of figures. We gratefully acknowledge the support of Dr Scott by a grant from the Special Trustees, Leeds General Infirmary, Leeds.

### References

BAGWELL, C.B. (1979). *Theory and application of DNA histogram analysis*. PhD thesis. University of Miami.

BAKER, S.J., FEARON, E.R., NIGRO, J.M. & 9 others (1989). Chromosome 17 deletions and p53 gene mutations in colorectal carcinomas. *Science*, **244**, 217.

BEART, R.W., MELTON, L.J., MARUTA, M., DOCKERTY, M.B., FRYDENBERG, H.B. & O'FALLON, W.M. (1983). Trends in right and left sided colon cancer. *Dis. Col. Rect.*, **26**, 393.

CATTORETTI, G., RILKE, F., ANDREOLA, S., D'AMATO, L. & DELIA, D. (1988). p53 expression in breast cancer. *Int. J. Cancer*, **41**, 178.

CRAWFORD, L.V., PIM, D.C. & LAMB, P. (1984). The cellular protein p53 in human tumours. *Mol. Biol. Med.*, **2**, 261.

DELATTRE, O., LAW, D.J., REMVIKOS, Y. & 7 others (1989). Multiple genetic alterations in distal and proximal colorectal cancer. *Lancet*, **ii**, 353.

ERISMAN, M.D., ROTHBERG, P.G., DIEHL, R.E., MORSE, C.C., SPANDORFER, J.M. & ASTRIN, S.M. (1985). Deregulation of c-*myc* gene expression in human colon carcinoma is not accompanied by amplification or rearrangement of the gene. *Mol. Cell Biol.*, **5**, 1969.

FINLAY, C.A., HINDS, P.W., TAN, T.H., ELIYAHU, D., OREN, M. & LEVINE, A.J. (1988). Activating mutations for transformation by p53 produce a gene product that forms an hsc 70-p53 complex with an altered half life. *Mol. Cell Biol.*, **8**, 531.

FINLAY, C.A., HINDS, P.W. & LEVINE, A.J. (1989). The p53 proto-oncogene can act as a suppressor of transformation. *Cell*, **57**, 1083.

HINDS, P., FINLAY, C. & LEVINE, A.J. (1989). Mutation is required to activate the p53 gene for cooperation with the ras oncogene and transformation. *J. Virol.*, **63**, 739.

IGGO, R., GATTER, K., BARTEK, J., LANE, D. & HARRIS, A.L. (1990). Increased expression of mutant forms of p53 oncogene in primary lung cancer. *Lancet*, **i**, 675.

KERN, S.E., TERSMETTE, K.W., FEARON, E.R. & 6 others (1989). Clinical associations with genetic alterations in colorectal carcinoma. *Proc. Ann. Meet. Am. Assoc. Cancer Res.*, **30**, A178.

LANE, D.P. & CRAWFORD, L.V. (1979). T antigen is bound to a host protein in SV40-transformed cells. *Nature*, **278**, 261.

MERCER, W.E., AVIGNOLO, C. & BASERGA, R. (1984). Role of the p53 protein in cell proliferation as studied by microinjection of monoclonal antibodies. *Mol. Cell Biol.*, **4**, 276.

MILNER, J. & MILNER, S. (1981). SV40-53K antigen: a possible role for 53K in normal cells. *Virology*, **112**, 785.

MOLLER JENSEN, O. (1984). Different age and sex relationship for cancer of subsites of the large bowel. *Br. J. Cancer*, **50**, 825.

QUIRKE, P., DIXON, M.F., CLAYDEN, A.D. & 4 others (1987). Prognostic significance of DNA aneuploidy and cell proliferation in rectal adenocarcinomas. *J. Pathol.*, **151**, 285.

REICH, N. & LEVINE, A.J. (1984). Growth regulation of a cellular tumour antigen, p53, in nontransformed cells. *Nature*, **308**, 199.

REMVIKOS, Y., LAURENT-PUIG, P., SALMON, R.J., FRELAT, G., DUTRILLAUX, B. & THOMAS, G. (1990). Simultaneous monitoring of p53 protein and DNA content of colorectal adenocarcinomas by flow cytometry. *Int. J. Cancer*, **45**, 450.

ROGEL, A., POPLIKER, M., WEBB, C.G. & OREN, M. (1985). p53 cellular tumour antigen: analysis of mRNA levels in normal adult tissues, embryos, and tumors. *Mol. Cell Biol.*, **5**, 2851.

ROTHBERG, P.G., SPANDORFER, J.M., ERISMAN, M.D. & 4 others (1985). Evidence that c-myc expression defines two genetically distinct forms of colorectal adenocarcinoma. *Br. J. Cancer*, **52**, 629.

THOMPSON, A.M., STEEL, C.M., CHETTY, V. & 5 others (1990). p53 gene mRNA expression and chromosome 17p allele loss in breast cancer. *Br. J. Cancer*, **61**, 74.

VAN DEN BERG, F.M., TIGGES, A.J., SCHIPPER, M.E.I., DEN HARTOG-JAGER, F.C.A., KROES, W.G.M. & WALBOOMERS, J.M.M. (1989). Expression of the nuclear oncogene p53 in colon tumours. *J. Pathol.*, **157**, 193.

VOGELSTEIN, B., FEARON, E.R., HAMITON, S.R. & 7 others (1988). Genetic alterations during colorectal tumor development. *NEJM*, **319**, 525.